# Cooperation of PD-1 and LAG-3 in the exhaustion of CD4^+^ and CD8^+^ T cells during bovine leukemia virus infection

**DOI:** 10.1186/s13567-018-0543-9

**Published:** 2018-06-19

**Authors:** Tomohiro Okagawa, Satoru Konnai, Asami Nishimori, Naoya Maekawa, Shinya Goto, Ryoyo Ikebuchi, Junko Kohara, Yasuhiko Suzuki, Shinji Yamada, Yukinari Kato, Shiro Murata, Kazuhiko Ohashi

**Affiliations:** 1 Department of Disease Control, Faculty of Veterinary Medicine, Hokkaido University, Sapporo, 060-0818 Japan; 2 Animal Research Center, Agriculture Research Department, Hokkaido Research Organization, Shintoku, 081-0038 Japan; 3 Division of Bioresources, Research Center for Zoonosis Control, Hokkaido University, Sapporo, 001-0020 Japan; 4 Global Station for Zoonosis Control, Global Institution for Collaborative Research and Education (GI-CoRE), Hokkaido University, Sapporo, 001-0020 Japan; 5 Department of Antibody Drug Development, Graduate School of Medicine, Tohoku University, Sendai, 980-8575 Japan; 6 Present Address: Laboratory of Immunology, Faculty of Pharmacy, Osaka Ohtani University, Tondabayashi, 584-8540 Japan

## Abstract

Bovine leukemia virus (BLV) is a retrovirus that infects B cells in cattle and causes bovine leukosis after a long latent period. Progressive exhaustion of T cell functions is considered to facilitate disease progression of BLV infection. Programmed death-1 (PD-1) and lymphocyte activation gene-3 (LAG-3) are immunoinhibitory receptors that contribute to T-cell exhaustion caused by BLV infection in cattle. However, it is unclear whether the cooperation of PD-1 and LAG-3 accelerates disease progression of BLV infection. In this study, multi-color flow cytometric analyses of PD-1- and LAG-3-expressing T cells were performed in BLV-infected cattle at different stages of the disease. The frequencies of PD-1^+^LAG-3^+^ heavily exhausted T cells among CD4^+^ and CD8^+^ T cells was higher in the blood of cattle with B-cell lymphoma over that of BLV-uninfected and BLV-infected cattle without lymphoma. In addition, blockade assays of peripheral blood mononuclear cells were performed to examine whether inhibition of the interactions between PD-1 and LAG-3 and their ligands by blocking antibodies could restore T-cell function during BLV infection. Single or dual blockade of the PD-1 and LAG-3 pathways reactivated the production of Th1 cytokines, interferon-γ and tumor necrosis factor-α, from BLV-specific T cells of the infected cattle. Taken together, these results indicate that PD-1 and LAG-3 cooperatively mediate the functional exhaustion of CD4^+^ and CD8^+^ T cells and are associated with the development of B-cell lymphoma in BLV-infected cattle.

**Electronic supplementary material:**

The online version of this article (10.1186/s13567-018-0543-9) contains supplementary material, which is available to authorized users.

## Introduction

Bovine leukemia virus (BLV) is a member of the genus *Deltaretrovirus* (subfamily *Orthoretrovirinae*, family *Retroviridae*) and is genetically related to human T-cell leukemia virus type 1 [1]. BLV infects B cells in cattle and is integrated into the host genome as a provirus [2, 3]. The majority (around 70%) of BLV infections in cattle remain clinically asymptomatic, referred to as aleukemic (AL). However, up to 30% of infected cattle develop persistent lymphocytosis (PL), characterized by non-malignant polyclonal expansion of IgM^+^CD5^+^ B cells, the majority of which harbor BLV provirus. After a long latent period, less than 5% of infected cattle develop malignant B-cell lymphoma in various lymph nodes, so-called enzootic bovine leukosis (EBL) [4]. During BLV infection in cattle, T-cell response is known to be suppressed in PL and EBL animals, but not in AL animals [5–12]. Suppression of T-cell proliferation in response to BLV antigens and Th1 cytokine production are observed in PL and EBL cattle and considered to be associated with disease progression [7–12].

To develop strategies to induce effective immune responses to BLV infection, previous studies have investigated the mechanism responsible for T-cell exhaustion [13–16]. A previous report has shown that the immunoinhibitory receptor programmed death-1 (PD-1) is upregulated in CD4^+^ and CD8^+^ T cells and is involved in the exhaustion of T-cell functions in BLV-infected cattle bearing B-cell lymphoma [13]. Further studies have confirmed the correlation between upregulation of lymphocyte activation gene-3 (LAG-3) on CD4^+^ and CD8^+^ T cells and disease progression in BLV infection [14, 15]. Respective ligands of PD-1 and LAG-3, programmed death-ligand 1 (PD-L1) and major histocompatibility complex class II (MHC II), were upregulated in B cells including BLV-infected B cells in BLV-infected cattle [14–18]. We therefore hypothesized that PD-1 and LAG-3 cooperatively contribute to the development of T-cell exhaustion during disease progression of BLV infection. According to evidences from mouse models and human patients with chronic infections, PD-1^+^LAG-3^+^ T cells is heavily exhausted functionally and strongly associated with progression of several chronic infections [19].

However, our previous studies on BLV infection were analyzed expression of PD-1 and LAG-3 separately in independent experiments [13–15], thereby it was unknown whether PD-1 and LAG-3 express in the same T-cell populations and play immunosuppressive roles cooperatively during BLV infection. In addition, LAG-3 expression has been investigated in AL and PL cattle, but not in EBL animals yet [14, 15]. To unravel the immunosuppression mediated by LAG-3 during the development of bovine leukemia, expression level of the molecule should be investigated in EBL animals. Multi-color flow cytometric analysis detecting PD-1^+^LAG-3^+^ T cells requires sensitive monoclonal antibodies (mAbs) against bovine immunoinhibitory molecules. Thus, we have established anti-bovine PD-1 [13], anti-bovine PD-L1 [20], and anti-bovine LAG-3 mAbs [21]. In this paper, therefore, the dual expression of PD-1 and LAG-3 on T cells were first analyzed among BLV-infected cattle at different stages of the disease.

Moreover, blockade of the PD-1/PD-L1 and LAG-3/MHC II interactions using cross-reactive polyclonal antibodies increase Th1 cytokine responses and decrease BLV proviral load ex vivo in our previous reports [15, 16]. However, binding activity of the polyclonal antibodies was very weak and the blockade effect of the immunoinhibitory pathways seems to be underestimated in the previous study [16]. To address this problem, in this study, we tested the immunomodulatory activity of the novel sensitive mAbs against bovine PD-L1 and LAG-3 established in recent studies [20, 21] by ex vivo T-cell blockade assays using peripheral blood mononuclear cells (PBMCs) of BLV-infected cattle. This study will be helpful to estimate the therapeutic potential of PD-1 and LAG-3 blockade for BLV infection.

## Materials and methods

### Samples from cattle and BLV diagnosis

Blood samples collected from cattle (Holstein–Friesian and Japanese black) in dairy and beef farms in Japan between 2014 and 2016. Informed consent was obtained from all owners of the animals used in the study. BLV infection was diagnosed at Graduate School of Veterinary Medicine, Hokkaido University. Briefly, genomic DNA was extracted from whole blood using the Wizard Genomic DNA Purification kit (Promega, Madison, WI, USA). BLV was detected by nested PCR targeting the BLV long terminal repeat region (LTR) using the following primers: BLV-LTR1 5′-TGT ATG AAA GAT CAT GCC GAC-3′ and BLV-LTR533 5′-AAT TGT TTG CCG GTC TCT-3′ for the initial PCR, and BLV-LTR256 5′-GAG CTC TCT TGC TCC CGA GAC-3′ and BLV-LTR453 5′-GAA ACA AAC GCG GGT GCA AGC CAG-3′ for the second PCR [22]. The number of lymphocytes in the blood was counted using an automatic hematology analyzer Celltac α (Nihon Kohden, Tokyo, Japan). BLV-positive cattle were classified as AL or PL according to the lymphocyte counts as follows: AL, < 10 000 cells/μL of blood; PL, ≥ 10 000 cells/μL of blood. Blood samples from cattle clinically diagnosed as bovine leukosis were confirmed as B-cell lymphoma by phenotypic analysis of tumor cells using flow cytometry as described previously [13, 23].

### Cell preparation

PBMCs were purified from the blood samples using density gradient centrifugation on Percoll (GE Healthcare, Buckinghamshire, England, UK), washed three times with phosphate-buffered saline (PBS), and suspended in PBS.

### Flow cytometric analysis of PD-1 and LAG-3 expression

Six-color analysis of PD-1- and LAG-3-expressing T cells was performed using fresh PBMCs obtained from the animals with various stages of the infection. PBMCs were incubated in PBS containing 10% goat serum (Sigma–Aldrich, St. Louis, MO, USA) at room temperature for 15 min to prevent nonspecific reactions. Cells were then washed and stained with anti-PD-1 monoclonal antibody (mAb) (5D2, rat IgG_2a_) [13] or rat IgG_2a_ isotype control (R35-95, BD Biosciences, San Jose, CA, USA) for 20 min at room temperature, washed with PBS containing 1% bovine serum albumin (BSA; Sigma–Aldrich), and labeled with APC-conjugated anti-rat Ig antibody (Southern Biotech, Birmingham, AL, USA) for 20 min at room temperature. Cells were then washed and stained with anti-LAG-3 mAb (71-2D8, rat IgG_1_) [21] or rat IgG_1_ isotype control (R3-34, BD Biosciences) for 20 min at room temperature. Finally, cells were washed and incubated with anti-CD4-FITC mAb (CC8; Bio-Rad, Hercules, CA, USA) or anti-CD8-FITC mAb (CC63, Bio-Rad) in the presence of anti-TCR1-N24-APC/Cy7 mAb (anti-TCR δ chain; GB21A; Washington State University Monoclonal Antibody Center, Pullman, WA, USA), anti-CD3-PerCp/Cy5.5 mAb (MM1A; Washington State University Monoclonal Antibody Center), anti-IgM-PE/Cy7 mAb (IL-A30; Bio-Rad), and PE-conjugated anti-rat IgG_1_ mAb (G17E7; Southern Biotech) for 20 min at room temperature. mAbs GB21A, MM1A, and IL-A30 were conjugated with APC/Cy7, PerCp/Cy5.5, and PE/Cy7, respectively, using Lightning-Link Conjugation Kits (Innova Biosciences, Cambridge, England, UK). Cells were then washed and immediately analyzed by FACS Verse (BD Biosciences) and FCS Express 4 (De Novo Software, Glendale, CA, USA). The primary antibodies used in this experiment are shown in Table 1. More than 100 000 lymphocytes were analyzed among the samples.

**Table 1:** **Primary antibodies used in flow cytometric analysis of this paper**

Target	Isotype	Clone	Source (reference)	Fluorochrome	Conjugation or labeling
Flow cytometric analysis of PD-1 and LAG-3					
CD4	Mouse IgG_2a_	CC8	Bio-Rad	FITC	(Conjugated primary antibody)
CD8	Mouse IgG_2a_	CC63	Bio-Rad	FITC	(Conjugated primary antibody)
CD3	Mouse IgG_1_	MM1A	WSU monoclonal Antibody Center	PerCp/Cy5.5	Lightning-Link PerCp/Cy5.5 Conjugation Kit (Innova Biosciences)
IgM	Mouse IgG_1_	IL-A30	Bio-Rad	PE/Cy7	Lightning-Link PE/Cy7 Conjugation Kit (Innova Biosciences)
TCR1-N24 (δ chain)	Mouse IgG_2b_	GB21A	WSU monoclonal Antibody Center	APC/Cy7	Lightning-Link APC/Cy7 Conjugation Kit (Innova Biosciences)
PD-1	Rat IgG_2a_	5D2	In house [13]	APC	APC-conjugated anti-rat Ig antibody (Southern Biotech)
LAG-3	Rat IgG_1_	71-2D8	In house [21]	PE	PE-conjugated anti-rat IgG_1_ antibody (Southern Biotech)
Flow cytometric analysis of IFN-γ/TNF-α-producing T cells (intracellular staining)					
CD4	Mouse IgG_2a_	CC8	Bio-Rad	FITC	(Conjugated primary antibody)
CD8	Mouse IgG_2a_	CC63	Bio-Rad	PerCp/Cy5.5	Lightning-Link PerCp/Cy5.5 Conjugation Kit (Innova Biosciences)
TCR1-N24 (δ chain)	Mouse IgG_2b_	GB21A	WSU monoclonal Antibody Center	Alexa Fluor 647	Zenon Alexa Fluor 647 Mouse IgG_2b_ Labeling Kit (Thermo Fisher Scientific)
IFN-γ	Mouse IgG_1_	CC302	Bio-Rad	PE	(Conjugated primary antibody)
TNF-α	Mouse IgG_2b_	CC328	Bio-Rad	APC/eFluor780	Streptavidin APC/eFluor780 (eBioscience)

FITC: fluorescein isothiocyanate, PerCp: peridinin-chlorophyll-protein complex, Cy: cyanin, PE: phycoerythrin, APC: allophycocyanin.

### PBMC blockade assay and IFN-γ ELISA

To determine whether the blockade of the PD-1/PD-L1 and LAG-3/MHC II interactions alter BLV-specific IFN-γ responses, PBMCs prepared from BLV-infected cattle were cultured with blocking mAbs anti-PD-L1 (4G12, rat IgG_2a_) [20], anti-LAG-3 (71-2D8, rat IgG_1_) [21], or rat IgG (Sigma–Aldrich) in the presence of heat-inactivated supernatant (2%) of BLV-infected fetal lamb kidney cells (FLK-BLV) at 37 °C with 5% CO_2_ for 6 days. FLK-BLV supernatant contained BLV antigens to stimulate BLV-specific T cells in PBMC cultures as described previously [9]. The heat-inactivated supernatant (2%) of BLV-uninfected fetal lamb kidney cells (FLK) was used as a negative control antigen. All cell cultures were grown in 96-well round-bottomed plates (Corning Inc., Corning, NY, USA) containing 1 × 10^6^ PBMCs in 250 μL RPMI 1640 medium (Sigma–Aldrich) supplemented with 10% heat-inactivated fetal bovine serum (Cansera International, Etobicoke, ON, Canada), 200 IU/mL penicillin, 200 μg/mL streptomycin, and 0.01% v/v l-glutamine (Thermo Fisher Scientific, Waltham, MA, USA). Culture supernatants were then harvested and stored at −20 °C for IFN-γ ELISA. IFN-γ concentrations in the supernatants were determined using a bovine IFN-γ ELISA (Mabtech, Nacka Strand, Sweden) performed in duplicate according to the manufacturer’s protocol.

### PBMC blockade assay and flow cytometric analysis of IFN-γ/TNF-α-producing T cells

To examine the effects of blocking mAbs on BLV-specific T-cell responses, in vitro blockade assay was performed. PBMCs were cultivated with 10 μg/mL of blocking mAbs anti-PD-L1 (4G12, rat IgG_2a_) [20], anti-LAG-3 (71-2D8, rat IgG_1_) [21], or rat IgG (Sigma–Aldrich) in the presence of 2% heat-inactivated FLK-BLV supernatant and 1 μg/mL of agonist mAbs anti-CD3 (MM1A; Washington State University Monoclonal Antibody Center) and anti-CD28 (CC220; Bio-Rad) at 37 °C with 5% CO_2_ for 18 h. 2% heat-inactivated FLK supernatant was used as a negative control antigen. All cell cultures were grown in 96-well round-bottomed plates (Corning Inc.) containing 1 × 10^6^ PBMCs in 250 μL RPMI 1640 medium (Sigma–Aldrich) supplemented as described above. BFA (10 μg/mL; Sigma–Aldrich) was added for the final 6 h to enhance intracellular cytokine staining signals by blocking transport processes. Cultured PBMCs were collected and incubated in PBS containing 10% goat serum as described above. Cells were then washed and stained with anti-CD4-FITC mAb (CC8; Bio-Rad), anti-CD8-PerCp/Cy5.5 mAb (CC63; Bio-Rad), and anti-TCR1-N24-Alexa Fluor 647 mAb (anti-TCR δ chain; GB21A; Washington State University Monoclonal Antibody Center) for 30 min at 4 °C. CC63 was conjugated with PerCp/Cy5.5 using Lightning-Link Antibody Labeling Kit (Innova Biosciences, Cambridge, England, UK). GB21A was prelabeled with Zenon Alexa Fluor 647 Mouse IgG_2b_ Labeling Kit (Thermo Fisher Scientific). After surface staining, cells were fixed and permeabilized using FOXP3 Fix/Perm kit (BioLegend, San Diego, CA, USA) according to the manufacturer’s protocol. Cells were then stained with anti-IFN-γ-PE (CC302; Bio-Rad) and anti-TNF-α-Biotin (CC328; Bio-Rad) for 30 min at 4 °C. Cells were then washed and incubated with streptavidin APC/eFluor780 (eBioscience, San Diego, CA) for 30 min at 4 °C. Finally, cells were washed and analyzed immediately using FACS Verse (BD Biosciences) and FCS Express 4 (De Novo Software). The primary antibodies used in this experiment are shown in Table 1. More than 10 000 lymphocytes were analyzed among the samples.

### Statistics

Significant differences in the frequency of each T-cell subset in expression analyses between groups were identified using Kruskal–Wallis test. A Friedman test was used to compare the IFN-γ production and the frequency of each cytokine-producing T-cell subpopulation between groups in blockade assays. All statistical tests were performed using GraphPad Prism 6 (GraphPad Software, San Diego, CA, USA). Differences were considered statistically significant when *P* < 0.05.

## Results

### Increased frequencies of heavily exhausted CD4^+^ and CD8^+^ T cells in BLV-infected cattle with B-cell lymphoma

**Figure 1:**
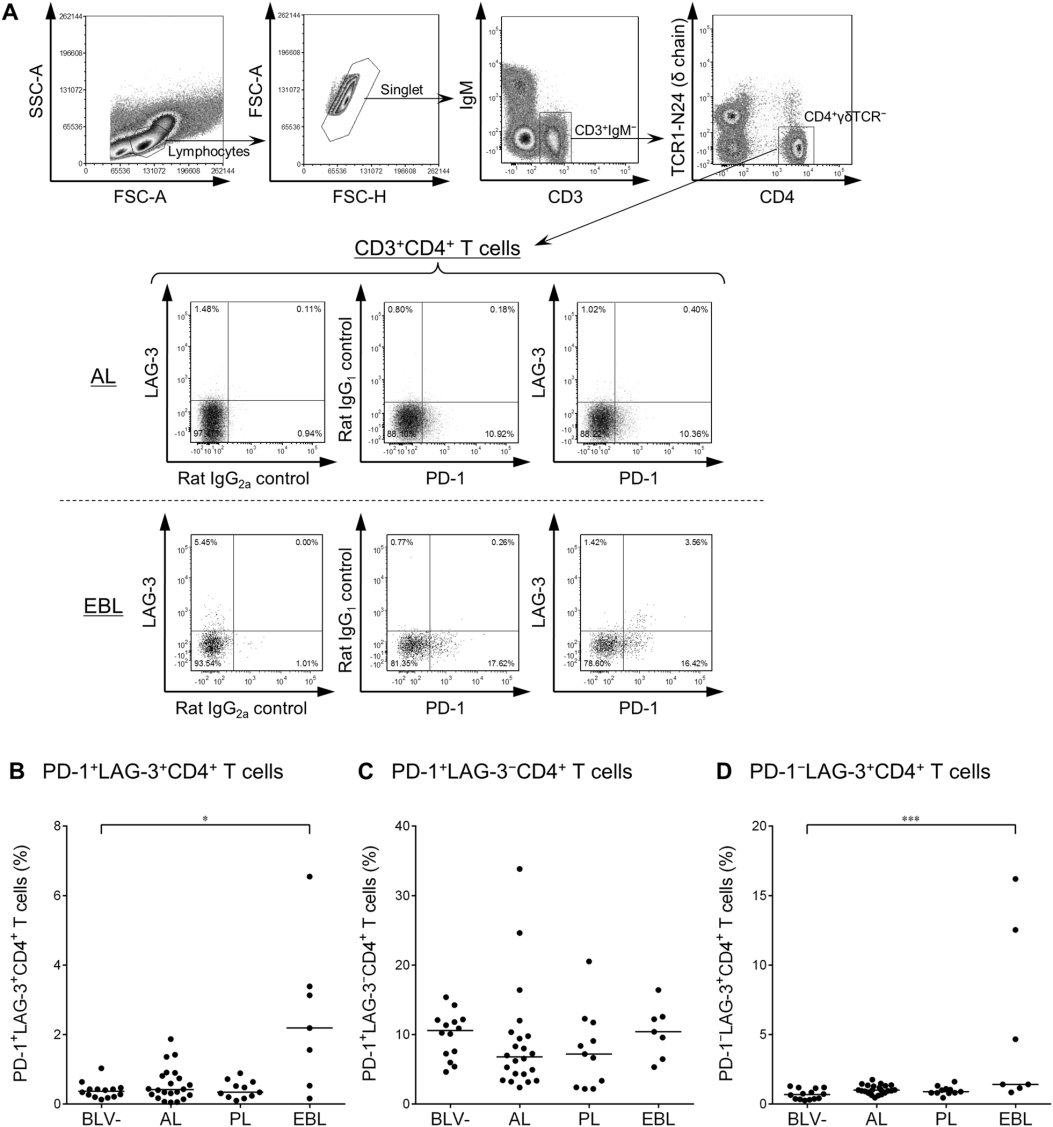
**Expression of PD-1 and LAG-3 on CD4**^**+**^ **T cells in BLV-infected cattle. A** Gating strategy and representative dot plots for expression analyses of PD-1 and LAG-3 on IgM^−^CD3^+^CD4^+^γδTCR^−^ T cells from peripheral blood of BLV-infected cattle (AL and EBL). Values in the quadrants indicate percentages of cells. Percentages of PD-1^+^LAG-3^+^CD4^+^ T cells (**B**), PD-1^+^LAG-3^−^CD4^+^ T cells (**C**), and PD-1^−^LAG-3^+^CD4^+^ T cells (**D**) in CD3^+^CD4^+^ T-cell population in peripheral blood from BLV-uninfected (BLV − ; *n* = 15), AL (*n* = 22), PL (*n* = 11), and EBL cattle (*n* = 7). Bars indicate group median percentage. Significant differences between each group were determined using a Kruskal–Wallis test, where *P* < 0.05 and *P* < 0.001, indicated by asterisks (* and ***, respectively).

**Figure 2:**
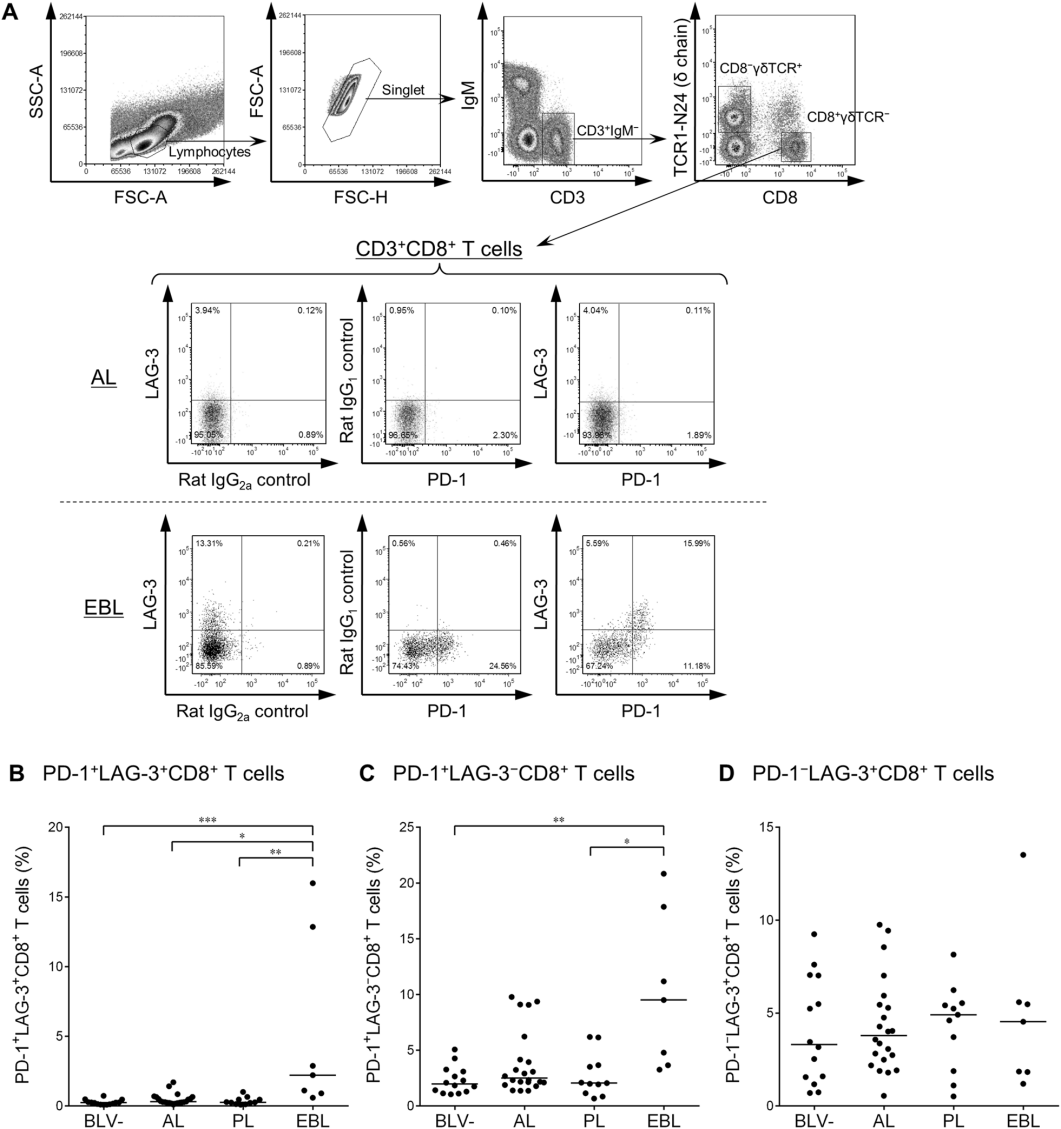
**Expression of PD-1 and LAG-3 on CD8**^**+**^ **T cells in BLV-infected cattle. A** Gating strategy and representative dot plots for expression analyses of PD-1 and LAG-3 on IgM^−^CD3^+^CD8^+^γδTCR^−^ T cells from peripheral blood of BLV-infected cattle (AL and EBL). Values in the quadrants indicate percentages of cells. Percentages of PD-1^+^LAG-3^+^CD8^+^ T cells (**B**), PD-1^+^LAG-3^−^CD8^+^ T cells (**C**), and PD-1^−^LAG-3^+^CD8^+^ T cells (**D**) in CD3^+^CD8^+^ T-cell population in peripheral blood from BLV − (*n* = 15), AL (*n* = 22), PL (*n* = 11), and EBL cattle (*n* = 7). Bars indicate group median percentage. Significant differences between each group were determined using a Kruskal–Wallis test, where *P* < 0.05, *P* < 0.01, and *P* < 0.001, indicated by asterisks (*, **, and ***, respectively).

**Figure 3:**
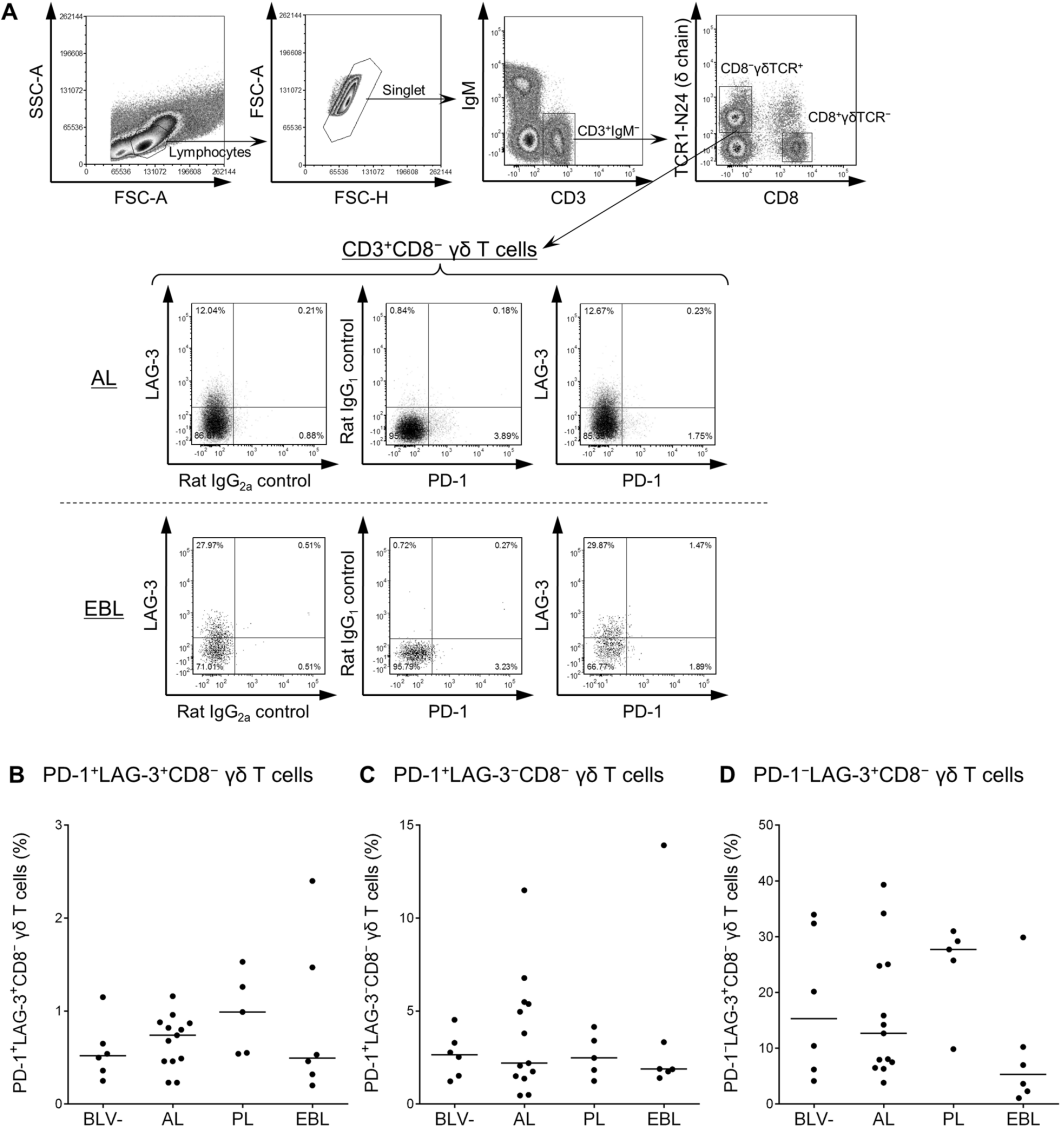
**Expression of PD-1 and LAG-3 on γδ T cells in BLV-infected cattle. A** Gating strategy and representative dot plots for expression analyses of PD-1 and LAG-3 on IgM^−^CD3^+^CD8^−^γδTCR^+^ T cells from peripheral blood of BLV-infected cattle (AL and EBL). Values in the quadrants indicate percentages of cells. Percentages of PD-1^+^LAG-3^+^CD8^−^ γδ T cells (**B**), PD-1^+^LAG-3^−^CD8^−^ γδ T cells (**C**), and PD-1^−^LAG-3^+^CD8^−^ γδ T cells (**D**) in CD3^+^CD8^−^ γδ T-cell population in peripheral blood from BLV − (*n* = 6), AL (*n* = 13), PL (*n* = 5), and EBL cattle (*n* = 6). Bars indicate group median percentage. Significant differences between each group were determined using a Kruskal–Wallis test.

Earlier studies have shown that immunoinhibitory receptors such as PD-1 and LAG-3 were upregulated and play an immunomodulatory role in T-cell exhaustion during BLV infection in cattle [13–15]. We therefore hypothesized that disease progression of BLV-infected cattle is associated with expansion of PD-1^+^LAG-3^+^ heavily exhausted T cells. To test this hypothesis, the cell-surface expression of PD-1 and LAG-3 on CD4^+^, CD8^+^, and γδ T cells was investigated by multi-color flow cytometry of PBMCs isolated from BLV-infected cattle in different disease stages (AL, PL, and EBL). As shown in Figures 1A, 2A, and 3A, CD4^+^γδTCR^−^, CD8^+^γδTCR^−^, or CD8^−^γδTCR^+^ T cells were gated in CD3^+^IgM^−^ lymphocytes and then analyzed for expression of PD-1 and LAG-3. The percentage of PD-1^+^LAG-3^+^CD4^+^ T cells was significantly larger in the EBL animals than in the BLV-uninfected animals (Figure 1B). In the CD8^+^ T cell subset, the frequency of PD-1^+^LAG-3^+^CD8^+^ T cells was significantly higher in the EBL group than in the other BLV-infected and BLV-uninfected groups (Figure 2B). No remarkable changes were observed in the populations of PD-1^+^LAG-3^−^CD4^+^ and PD-1^−^LAG-3^+^CD8^+^ T cells of EBL animals (Figures 1B and 2C), but the percentages of PD-1^−^LAG-3^+^CD4^+^ and PD-1^+^LAG-3^−^CD8^+^ T cells were also significantly higher in the EBL group (Figures 1C and 2B). In contrast, no significant differences were observed in any analyzed subsets of CD8^−^ γδ T cells among the tested groups (Figures 3B–D), although the number of samples available for this analysis was small. To evaluate the status of all the tested EBL animals and its contribution to the phenotype of T cells, the individual datasets were added as Additional file 1. No tested parameters including age of onset, lymphocyte counts, proviral load, tumor cell type, and B-cell occupancy in blood were significantly correlated with PD-1^+^LAG-3^+^CD4^+^ and PD-1^+^LAG-3^+^CD8^+^ T-cell populations of the EBL animals. Taken together, the frequency of heavily exhausted T cells (PD-1^+^LAG-3^+^) in the population of peripheral CD4^+^ and CD8^+^ T cells increases in BLV-infected cattle bearing tumor.

### Reactivation of IFN-γ production by PD-1 and LAG-3 blockade

**Figure 4:**
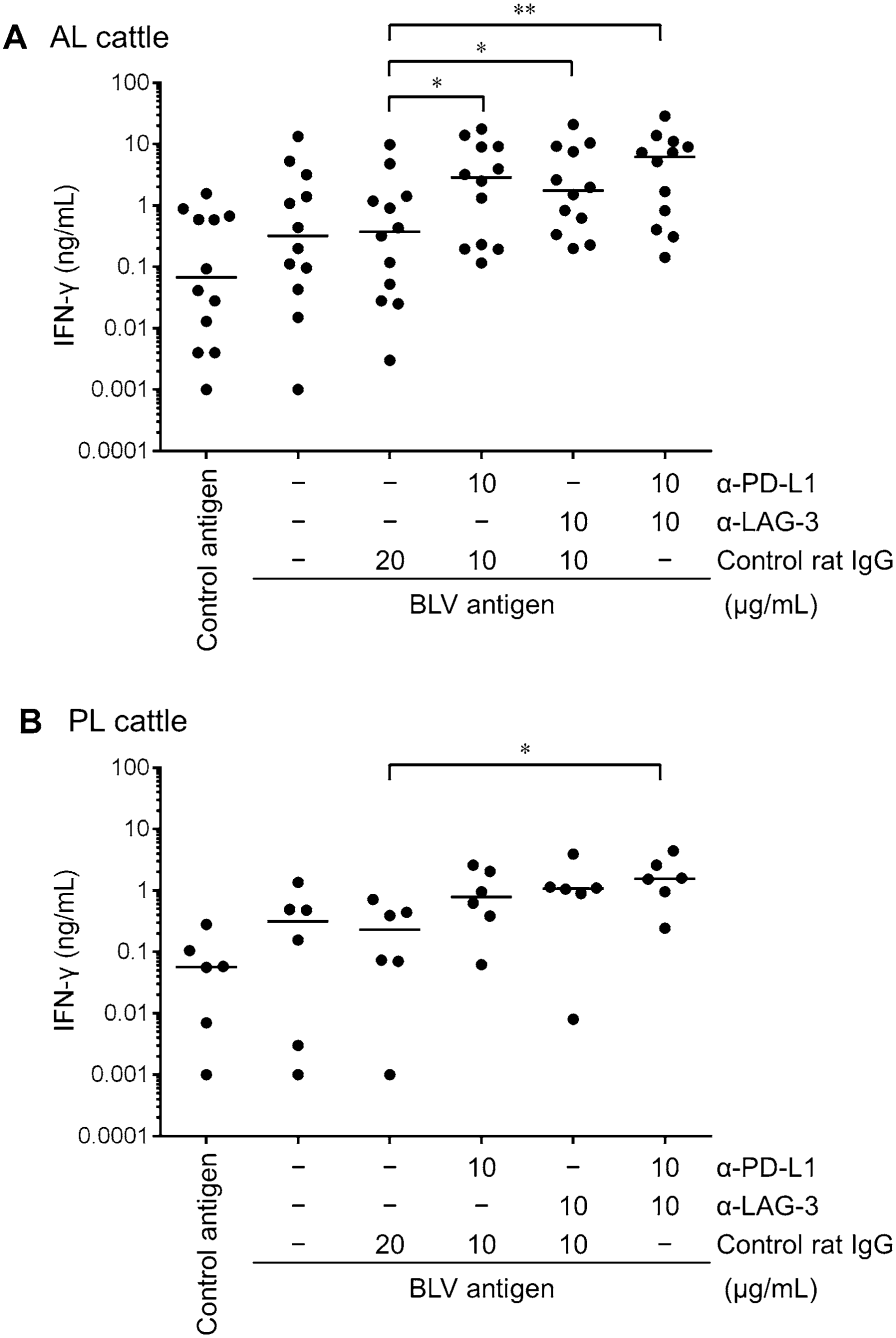
**Effect of PD-1/PD-L1 and LAG-3/MHC II blockade on BLV-specific IFN-γ response.** PBMCs of BLV-infected AL (**A**; *n* = 12) and PL cattle (**B**; *n* = 6) were cultured with blocking mAbs (anti-PD-L1 and anti-LAG-3 mAbs; 10 μg/mL) or rat IgG control in the presence of FLK-BLV antigen (2%) for 6 days. IFN-γ production for each animal was measured by ELISA in duplicate. Bars indicate group median response. Significant differences between each group were determined using Friedman test, where *P* < 0.05 and *P* < 0.01, indicated by asterisks (* and **, respectively).

Several studies have shown that Th1 response including IFN-γ production is impaired during disease progression of BLV infection, especially in PL cattle [7–12]. In this study, to determine whether BLV-specific IFN-γ response was restored by the blockade using novel anti-PD-L1 [20] and anti-LAG-3 mAbs [21], blockade assay were performed using PBMCs of BLV-infected AL and PL cattle. Single blockade using either anti-PD-L1 or anti-LAG-3 mAbs showed significantly increased production of IFN-γ from PBMCs of AL animals in response to BLV antigen (7.5- and 4.6-fold over control IgG, respectively) (Figure 4A). In contrast, IFN-γ production in PBMCs of PL animals were not significantly increased by PD-L1 or LAG-3 blockade, though the IFN-γ concentrations tended to be increased by each blockade (3.3- and 4.6-fold over control IgG, respectively) (Figure 4B). Notably, dual blockade by anti-PD-L1 and anti-LAG-3 mAbs significantly enhanced IFN-γ response to BLV antigen in PBMCs of both AL and PL groups (16.4- and 6.7-fold over control IgG, respectively) (Figures 4A and B). There results show that BLV-specific IFN-γ response can be reactivated by the in vitro blockade of PD-1/PD-L1 and LAG-3/MHC II interactions in BLV-infected cattle, even with progressed disease.

### Reactivation of Th1 cytokine production from T cells by PD-1 and LAG-3 blockade

**Figure 5:**
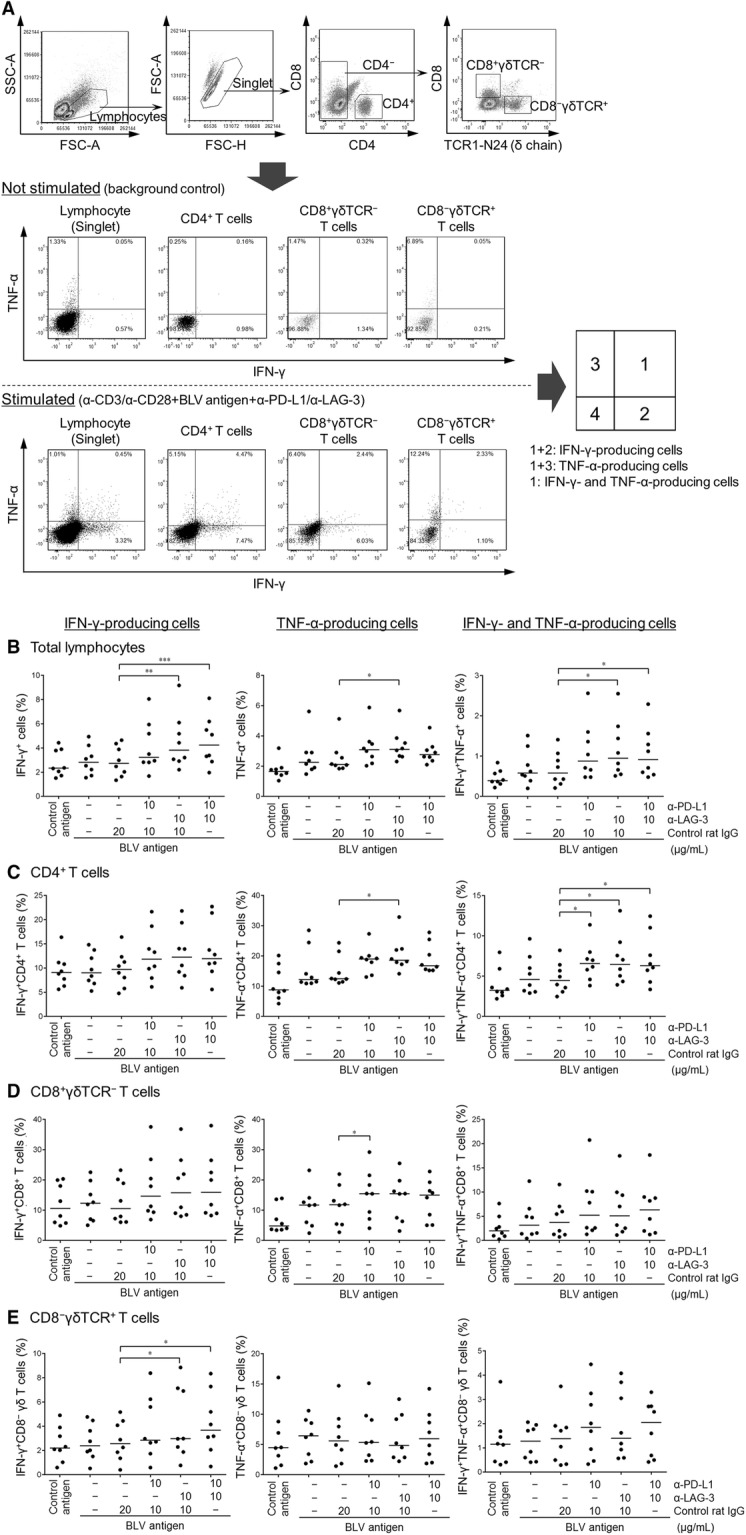
**Effect of PD-1/PD-L1 and LAG-3/MHC II blockade on Th1 cytokine production in BLV-specific T cells.** PBMCs (*n* = 8; AL *n* = 6, PL *n* = 2) were cultivated with 10 µg/mL each of blocking mAbs (anti-PD-L1 and anti-LAG-3 mAbs; 10 μg/mL) or rat IgG control in the presence of FLK-BLV antigen (2%) and anti-CD3 and anti-CD28 agonist mAbs (1 µg/mL each) for 18 h. **A** Gating strategy and representative dot plots for expression analyses of IFN-γ and TNF-α on total lymphocytes, CD4^+^ T cells, CD8^+^γδTCR^−^ T cells, and CD8^−^γδTCR^+^ T cells from peripheral blood of an AL animal. Values in the quadrants indicate percentages of cells. Percentages of IFN-γ^+^ (left), TNF-α^+^ (center), IFN-γ^+^TNF-α^+^ cells (right) among total lymphocytes (**B**), CD4^+^ T cells (**C**), CD8^+^γδTCR^−^ T cells (**D**), and CD8^−^γδTCR^+^ T cells (**E**) in PBMCs treated with blocking mAbs. Bars indicate group median response. Significant differences between each group were determined using Friedman test, where *P* < 0.05, *P* < 0.01, and *P* < 0.001, indicated by asterisks (*, **, and ***, respectively).

Because we observed the enhancement of IFN-γ response to BLV antigen by the blockade of the immunoinhibitory pathways, it was important to determine which subsets of T cells were induced to produce Th1 cytokines in PBMC blockade. To address this issue, PBMC blockade assay was performed in the presence of BLV antigen and agonist mAbs to CD3 and CD28, and then cells producing IFN-γ and TNF-α were analyzed in each T-cell subset by flow cytometry (AL *n* = 6, PL *n* = 2). As shown in Figure 5A, CD4^+^, CD8^+^γδTCR^−^, or CD8^−^γδTCR^+^ T cells were gated in lymphocytes and then analyzed for expression of IFN-γ and TNF-α. Consistent with the data of Figure 4, the frequency of IFN-γ producing cells in total lymphocytes significantly increased by the dual blockade using anti-PD-L1 and anti-LAG-3 mAbs (Figure 5B, left). Additionally, LAG-3 blockade significantly enhanced TNF-α production in lymphocytes (Figure 5A, center). Remarkably, IFN-γ/TNF-α double producers are activated by anti-LAG-3 mAb alone and in combination with anti-PD-L1 mAb (Figure 5B, right). Phenotypic analyses of cytokine-producing T cells revealed that CD4^+^ and CD8^+^ T cells, but not CD8^−^ γδ T cells, were induced to produce the larger amounts of Th1 cytokines by the blockade of the PD-1 and LAG-3 pathways (Figures 5C–E). Single or dual treatment of anti-PD-L1 and anti-LAG-3 mAbs induced significant increases in the percentages of IFN-γ^+^TNF-α^+^CD4^+^ T cells (Figure 5C, right). TNF-α production in CD4^+^ and CD8^+^ T cells was significantly enhanced by single blockade using anti-PD-L1 or anti-LAG-3 mAb (Figures 5C and D, center). Unexpectedly, the frequencies of IFN-γ-producing CD8^−^ γδ T cells were significantly higher in cultures using anti-LAG-3 mAb with or without anti-PD-L1 mAb (Figure 5E, left). Overall, these results indicate that BLV-specific Th1 response can be reactivated by the blockade of the PD-1 and LAG-3 pathways in BLV-infected cattle.

## Discussion

Cases of BLV infection and BLV-induced EBL in cattle have been increasing in Japan [24, 25]. Nevertheless, there is neither effective treatment nor vaccination to control BLV infection, which is due in part to the lack of understanding of the immunological mechanisms leading to immune evasion. During BLV infection, especially in the PL and EBL stages, T-cell exhaustion characterized by impaired proliferation of BLV-specific T cells and downregulation of Th1 cytokines accelerates disease progression [7–12]. Previous studies have clarified the molecular mechanism responsible for this T-cell exhaustion, implicating immunoinhibitory receptors such as PD-1 and LAG-3 in this process [13–15]. PD-1 upregulation has been observed in CD4^+^ and CD8^+^ T cells in peripheral blood and lymph nodes of EBL cattle [13]. Expression level of LAG-3 upregulated on peripheral CD4^+^ and CD8^+^ T cells in PL cattle and correlated positively with lymphocytosis and negatively with IFN-γ expression in CD4^+^ T cells [15]. In the present study, we conducted immunophenotypic and functional analyses of PD-1- and LAG-3-expressing exhausted T cells in BLV-infected cattle with different stages of disease.

The immunophenotypic analyses have revealed that PD-1^+^LAG-3^+^ T cells are expanded in peripheral among CD4^+^ and CD8^+^ T cells during the EBL stage but not the PL stage. The tested EBL animals developed monoclonal or oligoclonal expansion of B cells in peripheral blood (data not shown); therefore, these observations indicate that PD-1^+^LAG-3^+^ T cells can be functionally exhausted and are associated with the tumor development. However, the contribution of these exhausted T-cell subsets to tumor phenotype and pathogenesis is not clear. In this study, no correlation was found between the frequency of exhausted T-cell subsets in EBL animals and age of the onset, disease parameters, or clinical signs (data not shown). Further immunophenotypic analysis of PD-1^+^LAG-3^+^ T cells in tumor lesions of EBL animals is desired to be conducted to address this issue.

In healthy cattle, PD-1 and LAG-3 are expressed mainly on CD4^+^ and CD8^+^ T cells, respectively [13, 21]. Indeed, PD-1^+^LAG-3^−^ and PD-1^−^LAG-3^+^ T cells were mainly observed in CD4^+^ and CD8^+^ T-cell subsets, respectively, of BLV-uninfected, AL and PL animals. In the EBL group, not only the median percentages of PD-1^+^LAG-3^+^CD4^+^ and PD-1^+^LAG-3^+^CD8^+^ T cells but also that of PD-1^−^LAG-3^+^CD4^+^ and PD-1^+^LAG-3^−^CD8^+^ T cells were larger than the other groups. These results imply that upregulations of LAG-3 on CD4^+^ T cells and PD-1 on CD8^+^ T cells and subsequent co-expressions with other inhibitory receptors are remarkable change during the development of B-cell lymphoma in BLV-infected cattle.

A large number of γδ T cells are found in T-cell population of cattle, comprising up to 80% of T cells in peripheral blood of young calves, and 10–20% of T cells in adult cattle [26, 27]. Bovine γδ T cells have cytolytic and cytokine secretory properties similar to those of αβ T cells, thereby playing key roles in adaptive immunity of cattle [26, 27]. PD-1 expression is known to impair IFN-γ production and cytolytic function in human γδ T cells [28]. In addition, a previous study demonstrated increase of LAG-3^+^ γδ T cells in peripheral blood of cattle during acute anaplasmosis [29]. In contrast, this study found that the expression levels of PD-1 and LAG-3 were not changed on CD8^−^ γδ T cells during BLV infection. Further experimentation is required to confirm immunomodulatory role of PD-1 and LAG-3 on bovine T cells, but it seems that these immunoinhibitory receptors might play the major role in CD4^+^ and CD8^+^ αβ T-cell subsets, not in γδ T cells, in cattle bearing lymphoma.

The mechanism underlying the upregulation of PD-1 and LAG-3 is unclear in BLV infection, but evidences in humans and mice suggest that expression of PD-1 and LAG-3 is dependent on massive antigen presentation and the cytokine environment for a long period during chronic diseases [19, 30, 31]. Therefore, period post-infection could be associated with viral load and tumor development as well as the upregulation of PD-1 and LAG-3. The period post-infection of each tested animal is unknown because these animals are naturally infected with BLV. The experimental infection is helpful to investigate kinetics of the immunoinhibitory molecules throughout development of the disease. Co-upregulation of PD-1 and LAG-3 was observed in CD8^+^ T cells specific for viral or tumor antigens during chronic infection with lymphocytic choriomeningitis virus and human ovarian cancer [32, 33]. In BLV-infected cattle, PD-1 and LAG-3 are expected to be co-expressed on BLV-specific T cells. Confirmation of the relationship between PD-1^+^LAG-3^+^ T cells and antigen-specific immunosuppression in BLV infection requires MHC-peptide tetramers for the detection of BLV-specific T cells by flow cytometry.

A previous study has shown that proliferation of CD4^+^ T cells in response to *gag*- and *env*-encoded BLV proteins is impaired in PL animals and is almost lost in EBL animals, which is a critical evidence of the exhaustion of BLV-specific CD4^+^ T cells [8]. In this study, blockade of the immunoinhibitory receptor-ligand interactions using our novel blocking mAbs, anti-PD-L1 [20] and anti-LAG-3 mAbs [21], resulted in successful reactivation of IFN-γ production in response to whole BLV antigen even in PL animals. According to flow cytometric analysis of IFN-γ- and TNF-α-producing T cells, blockade of either PD-1/PD-L1 or LAG-3 was sufficient to activate double cytokine-producing multifunctional CD4^+^ T cells. These results suggest that the dual blockade has a potential to prevent disease progression by restoring anti-viral immunity during BLV infection. However, no significant correlation between the blockade effects and clinical parameters (proviral load, lymphocyte counts, and B cell occupancy in blood) was identified in the tested animals (data not shown). Additionally, most of the expression analyses and blockade assays were performed separately. The data of PD-1 and LAG-3 expression is available only for four animals which PBMC samples were also tested for the blockade assay at the same time. Therefore, association between the frequency of PD-1^+^LAG-3^+^ T-cell subsets and the blockade outcomes is unclear. In this study, the blockade assays were also performed using PBMCs of EBL animals, but the number of T cells was so small that the level of IFN-γ production could not analyzed by IFN-γ ELISA and flow cytometry (data not shown). To evaluate the blockade effect in EBL animals, more sensitive methods to detect IFN-γ production from BLV-specific T cells are required, such as Enzyme-Linked ImmunoSpot assay with epitope peptides or immunogenic proteins, not whole viral protein [34, 35].

We have conducted immunophenotypic and functional analyses of exhausted T cells in various chronic infections in cattle, such as BLV infection, Johne’s disease, bovine anaplasmosis, and bovine mycoplasmosis [21, 29, 36, 37]. Findings in these studies reveal that T-cell exhaustion is caused by the upregulation of PD-1 and LAG-3 and presumably facilitates persistent infection and disease progression in cattle with chronic infections. BLV infection is one of the good models of infectious diseases causing T-cell exhaustion in cattle as well as the disease to be addressed.

Numbers of researchers and pharmaceutical companies have investigated antibody treatments that block these “immune checkpoint” in human, and anti-PD-1, anti-PD-L1, and anti-LAG-3 antibodies have been approved for various human cancers or evaluated on clinical trials with cancer patients [38, 39]. We recently established anti-bovine PD-1 and anti-bovine PD-L1 rat**–**bovine chimeric antibodies as candidate agents for blocking the PD-1/PD-L1 pathway in vivo [40, 41]. Administration of these chimeric antibodies to BLV-infected calves enhanced BLV-specific CD4^+^ T-cell proliferation and reduced BLV proviral load in vivo. Thus, targeting immunoinhibitory pathway is a significant strategy for the regulation of T-cell response to BLV infection in cattle. In order to establish novel immunotherapies against BLV infection, additional studies are required to confirm the mechanisms underlying immunomodulatory and anti-viral effects of blocking antibody treatment in BLV-infected cattle.

## Additional file

**Additional file 1.** **Disease status and T-cell phenotype of individual EBL animals tested in this study.** To evaluate the status of all the tested EBL cattle and its contribution to the phenotype of T cells, the individual data are shown as Additional file 1. The dataset includes age of onset, lymphocyte count in peripheral blood, BLV proviral load, tumor cell type, the percentage of B cells in peripheral blood, and the percentages of PD-1^+^LAG-3^+^ cells, PD-1^+^LAG-3^−^ cells, and PD-1^−^LAG-3^+^ cells in CD4^+^, CD8^+^, and γδTCR^+^ T-cell subset of seven EBL animals tested in this study. Materials and methods related to only this dataset are also shown in additional file.
