# Supplementary material for: Cooperation of PD-1 and LAG-3 in the exhaustion of CD4+ and CD8+ T cells during bovine leukemia virus infection
Source: Vet Res. 2018 Jun 19;49:50. doi: 10.1186/s13567-018-0543-9 (PMC6006750; doi:10.1186/s13567-018-0543-9)
Supplement: Supplementary file 1 — Additional file 1. Disease status and T-cell phenotype of individual EBL animals tested in this study. To evaluate the status of all the tested EBL cattle and its contribution to the phenotype of T cells, the individual data are shown as Additional file 1. The dataset includes age of onset, lymphocyte count in peripheral blood, BLV proviral load, tumor cell type, the percentage of B cells in peripheral blood, and the percentages of PD-1+LAG-3+ cells, PD-1+LAG-3− cells, and PD-1−LAG-3+ cells in CD4+, CD8+, and γδTCR+ T-cell subset of seven EBL animals tested in this study. Materials and methods related to only this dataset are also shown in additional file. [file 13567_2018_543_MOESM1_ESM.docx]

**Additional file 1** **Disease status and T-cell phenotype of individual EBL animals tested in this study.**

| Animal # | Age  (month) | Lymphocyte  (cells/μL) | Proviral load  (copies/50 ng) | Tumor cell type | B cells (% in lymphocyte) | % in CD4^+^ T cells | | | % in CD8^+^ T cells | | | % in CD8^−^ γδ T cells | | |
| --- | --- | --- | --- | --- | --- | --- | --- | --- | --- | --- | --- | --- | --- | --- |
|  |  |  |  |  |  | PD-1^+^LAG-3^+^ cells | PD-1^+^LAG-3^−^ cells | PD-1^−^LAG-3^+^ cells | PD-1^+^LAG-3^+^ cells | PD-1^+^LAG-3^−^ cells | PD-1^−^LAG-3^+^ cells | PD-1^+^LAG-3^+^ cells | PD-1^+^LAG-3^−^ cells | PD-1^−^LAG-3^+^ cells |
| EBL1 | 66 | 62 800 | 2,110 | IgM^+^ B cell | 62.20 | 6.55 | 10.40 | 16.20 | 2.88 | 9.51 | 13.52 | N/A | N/A | N/A |
| EBL2 | 68 | 78 000 | 6,284 | IgM^+^ B cell | 91.95 | 0.16 | 9.57 | 0.84 | 1.11 | 3.66 | 1.86 | 0.53 | 1.87 | 7.00 |
| EBL3 | 84 | N/A | 4,099* | IgM^−^ B cell | 94.13 | 0.53 | 12.21 | 1.16 | 2.21 | 20.83 | 1.83 | 0.46 | 1.75 | 3.64 |
| EBL4 | 26 | 79,600 | 9,887 | IgM^+^ B cell | 78.52 | 3.56 | 16.42 | 1.42 | 15.99 | 11.18 | 5.59 | 1.47 | 1.89 | 29.87 |
| EBL5 | 31 | 48,000 | 5,736 | IgM^+^ B cell | 92.99 | 1.56 | 5.33 | 4.67 | 0.60 | 4.79 | 1.20 | 0.20 | 3.33 | 1.07 |
| EBL6 | 39 | 16,500 | 8,206 | IgM^+^ B cell | 95.40 | 3.39 | 6.47 | 12.54 | 0.91 | 3.27 | 4.54 | 0.32 | 1.40 | 10.23 |
| EBL7 | 128 | N/A | 5,215* | IgM^−^ B cell | 70.09 | 2.19 | 12.58 | 1.42 | 12.86 | 17.87 | 5.48 | 2.40 | 13.92 | 2.29 |
| N/A: not available. *The quality of DNA extracted from blood samples was poor and the real-time PCR analysis was not available for these samples.  Alternatively, proviral load data of lymph nodes bearing tumor from same individuals were shown in the table just to confirm BLV infection of the animals. | | | | | | | | | | | | | | |

# Materials and methods

**Lymphoma phenotyping of blood and tissue samples**

Peripheral blood and tissue (lymph nodes) in cattle with lymphoma were collected from livestock hygiene centers and meat hygiene inspection centers in Japan. Peripheral blood mononuclear cells (PBMCs) were purified by density-gradient centrifugation (Percoll; GE Healthcare, Buckinghamshire, England, UK). Tumor samples were cut up with scissors into small pieces, and the single-cell suspension was collected and washed twice with phosphate-buffered saline. Genomic DNA was extracted from 1 to 5 × 10^6^ PBMCs or single cells from tissues using a Wizard Genomic DNA Purification Kit (Promega, Madison, WI, USA). Blood and tissue samples from cattle were confirmed as B-cell lymphoma by phenotypic analysis of tumor cells using flow cytometry as described previously [13, 23].

**Quantification of BLV proviral load**

To determine proviral loads in the Boch5D2-inoculated animal, BLV *tax* gene was measured by quantitative real-time PCR. Briefly, genomic DNA was extracted from 2 × 10^6^ PBMCs with a Wizard Genomic DNA Purification Kit (Promega). Amplification of the BLV *tax* gene was performed in a reaction mixture containing 5 µL of Cycleave PCR Reaction Mix (Takara Bio, Otsu, Japan), 0.5 µL of Probe/Primer Mix for BLV (Takara Bio), 1 µL of a DNA template, and 3.5 µL of RNase-Free Distilled Water (Takara Bio) with a LightCycler 480 system II (Roche Diagnostics, Mannheim, Germany). Serial dilution of BLV Positive Control (Takara Bio) was used to generate calibration curves to determine the copy number of the BLV *tax* gene. Each DNA sample was tested in triplicate, and the reported values are the mean numbers of copies per 50 ng of DNA. The concentration of DNA was measured by UV absorbance at 260 nm with a NanoDrop 8000 Spectrophotometer (Thermo Fisher Scientific, Waltham, MA, USA).

**Statistical analysis**

Correlations were analyzed using Spearman's rank correlation coefficient. All statistical tests were performed with GraphPad Prism 6 (GraphPad Software, San Diego, CA, USA). Differences were considered statistically significant when *P* < 0.05.
